# Multi-Shared-Task Self-Supervised CNN-LSTM for Monitoring Free-Body Movement UPDRS-III Using Wearable Sensors

**DOI:** 10.3390/bioengineering11070689

**Published:** 2024-07-07

**Authors:** Mustafa Shuqair, Joohi Jimenez-Shahed, Behnaz Ghoraani

**Affiliations:** 1 Department of Electrical Engineering and Computer Science, Florida Atlantic University, Boca Raton, FL 33431, USA; mshuqair2020@fau.edu; 2 Department of Neurology, Icahn School of Medicine at Mount Sinai, New York, NY 10029, USA; joohi.jimenez-shahed@mountsinai.org

**Keywords:** Parkinson’s disease, deep learning, self-supervised learning, wearable systems, health monitoring

## Abstract

The Unified Parkinson’s Disease Rating Scale (UPDRS) is used to recognize patients with Parkinson’s disease (PD) and rate its severity. The rating is crucial for disease progression monitoring and treatment adjustment. This study aims to advance the capabilities of PD management by developing an innovative framework that integrates deep learning with wearable sensor technology to enhance the precision of UPDRS assessments. We introduce a series of deep learning models to estimate UPDRS Part III scores, utilizing motion data from wearable sensors. Our approach leverages a novel Multi-shared-task Self-supervised Convolutional Neural Network–Long Short-Term Memory (CNN-LSTM) framework that processes raw gyroscope signals and their spectrogram representations. This technique aims to refine the estimation accuracy of PD severity during naturalistic human activities. Utilizing 526 min of data from 24 PD patients engaged in everyday activities, our methodology demonstrates a strong correlation of 0.89 between estimated and clinically assessed UPDRS-III scores. This model outperforms the benchmark set by single and multichannel CNN, LSTM, and CNN-LSTM models and establishes a new standard in UPDRS-III score estimation for free-body movements compared to recent state-of-the-art methods. These results signify a substantial step forward in bioengineering applications for PD monitoring, providing a robust framework for reliable and continuous assessment of PD symptoms in daily living settings.

## 1. Introduction

Accurate and objective biomedical monitoring systems for Parkinson’s disease (PD) are essential for disease assessment and personalized treatment. PD is a progressive neurological disorder primarily affecting older individuals, impacting both motor and non-motor functions [1]. Patients often experience troublesome motor complications such as tremors, rigidity, slow movement, and difficulty walking [2]. Managing motor complications requires therapeutic adjustments during clinical visits. The Unified Parkinson’s Disease Rating Scale Part III (UPDRS-III) is a traditional assessment tool neurologists use to evaluate the severity of PD motor complications during these visits. The UPDRS-III involves patients performing a series of tasks in a clinical setting, with the assessments guiding therapeutic decisions. However, these evaluations occur sporadically and may not precisely capture the ongoing status of a patient’s condition, potentially leading to inappropriate treatment levels with subsequent complications [3].

Recent advancements in sensing technologies and machine learning could enable continuous, objective monitoring of PD symptoms in real-time, paving the way for personalized treatment paradigms, especially those associated with PD symptoms [4,5,6]. These innovations could extend to home-based applications, providing a means for monitoring daily motor fluctuations and effective management of PD medications [7], mitigating frequent in-person clinical examinations and reducing costs with more convenient solutions.

Building on this premise, our paper is rooted in the evolving landscape of bioengineering and biosignal processing research that harnesses deep learning and wearable sensing technology capabilities to provide an innovative approach for estimating UPDRS-III scores in PD patients. Our approach uses data gathered from wrist and ankle sensors, which monitor patients’ movements throughout their daily activities. The proposed methodology leverages the synergistic capabilities of Multichannel Convolutional Neural Networks (CNNs) and Long Short-Term Memory (LSTM) networks alongside a novel approach to Self-supervised Learning (SSL). Our technical innovations and contributions are as follows:

- We introduce a multichannel CNN-LSTM framework designed to process and analyze raw gyroscope sensor data and their spectrogram representations in parallel. This architecture allows for the comprehensive extraction of spatial and temporal features from PD patients’ movement data, significantly enhancing the accuracy of UPDRS-III score estimation. Integrating 1D CNN models for raw signal processing and 2D CNN models for spectrogram analysis, coupled with LSTM networks for capturing long-term dependencies, represents a novel approach. This combination effectively addresses the complexities of PD symptom manifestation in sensor data, setting a new standard for precision in PD monitoring technologies.
- Our other novel contribution is extending the capabilities of SSL by introducing a Multi-shared-task SSL (M-SSL) strategy. This approach leverages unlabeled data to pre-train a multichannel CNN on various signal transformation recognition tasks, significantly improving the model’s ability to extract and learn meaningful features from PD motion data without human annotation. Implementing shared layers between the branches of the CNN for each transformation recognition task, based on the congruence of spectrograms and raw signals, introduces a novel mechanism for enhancing feature learning. This method’s ability to refine data representation and feature extraction without labeled data is a considerable advancement over traditional SSL applications in bioengineering.
- We methodologically configure the multichannel CNN-LSTM network, including specific convolutional blocks and LSTM layers, optimized through the Bayesian technique. This setup is tailored for the dual objectives of learning signal representations and estimating UPDRS-III scores, thus offering a robust foundation for capturing the full spectrum of PD symptoms. This innovative selection of convolutional kernel sizes, pooling layers, and dropout rates, alongside integrating LSTM layers for sequence modeling, enables precise UPDRS-III score estimation from complex sensor data.

Our approach addresses the gap in current technical signal processing methods—primarily, their limitations in providing continuous, accurate UPDRS-III estimation from everyday life activities. This could lead to improved treatment customization and patient care, indicating a step toward more tailored healthcare solutions for individuals with PD.

## 2. Related Work

Bioengineering research for PD management has increasingly utilized wearable sensors and machine learning algorithms to assess disease progression and symptom severity. Primary efforts have predominantly focused on overcoming algorithmic challenges and fostering innovations in signal analysis to quantify PD “symptoms” accurately. Studies in this area have addressed various aspects, including estimating UPDRS-III sub-scores. Some research has concentrated on quantifying bradykinesia [8,9,10,11], objectively assessing slowness of movement in PD patients.Others have focused on detecting tremors and estimating their severity [12,13,14,15], while some have targeted the estimation of dyskinesia severity, which involves involuntary movements as medication side effects [16,17,18].

Moving beyond PD symptom severity into the challenge of UPDRS-III score estimation, Nilashi et al. [19] explored feature extraction from wearable sensor data, utilizing incremental support vector regression for score prediction. Zhan et al. [20] developed a mobile Parkinson’s disease score (mPDS) system that establishes correlations with UPDRS-III scores through an ensemble of tasks assessing gait, balance, finger tapping, reaction time, and voice. The application of adaptive neuro-fuzzy inference systems for analyzing accelerometer and gyroscope data was investigated by Butt et al. [21], focusing on some MDS-UPDRS-III tasks while wearing two wearable sensing devices. In another study by Sotirakis et al. [22], six sensors were utilized to gather PD patients’ walking and postural sway data. Afterward, feature extraction was performed, followed by a random forest algorithm estimation.

Despite these advancements, several limitations persist within the field. A primary constraint is the requirement for patients to actively participate in UPDRS-III-specific tasks during assessments, which may not capture the full spectrum of symptom severity experienced during daily activities. Moreover, the heavy reliance on feature extraction techniques to estimate UPDRS-III scores risks missing nuanced yet clinically significant information in raw sensor data [23]. This necessitates specific tasks that elicit measurable symptoms, limiting assessments to discrete moments and interrupting the continuum of daily life, thus providing only episodic insights into symptom fluctuations. Researchers have investigated and analyzed raw sensor data for UPDRS-III score estimation to address this challenge. For example, Hssayeni et al.’s work employs a combination of hand-crafted features, raw temporal signals, and time-frequency representation analyzed through an ensemble of deep learning models, showing a significant improvement over task-dependent models for continuous UPDRS-III score monitoring [24]. Rehman et al. [25] focused on deep convolutional neural networks processing accelerometer sensor data at the lower back to collect gait data for estimating UPDRS-III scores during walking.

Building on prior research, our proposed work seeks to bridge these gaps by introducing a multichannel CNN-LSTM framework and a novel M-SSL methodology. Our approach is designed to overcome the limitations of task-specific assessments and the constraints of traditional feature extraction methods. By leveraging the capabilities of multichannel CNNs for parallel processing of raw sensor data and their spectrogram representations, combined with the sequential data processing strengths of LSTM networks, our methodology facilitates a more nuanced, continuous, and comprehensive assessment of PD symptoms. Furthermore, the innovative application of M-SSL enables our model to learn and extract meaningful features from unlabeled data, enhancing the model’s performance without requiring specific patient task participation. This represents a significant technical advancement over existing methods and offers a more accurate, unobtrusive, and continuous monitoring solution for PD management, addressing the critical need for a methodology that accurately reflects the daily symptom severity experienced by individuals with PD and paving the way for significant advancements in the quality of life for PD patients.

## 3. Materials and Methods

**Figure 1:**
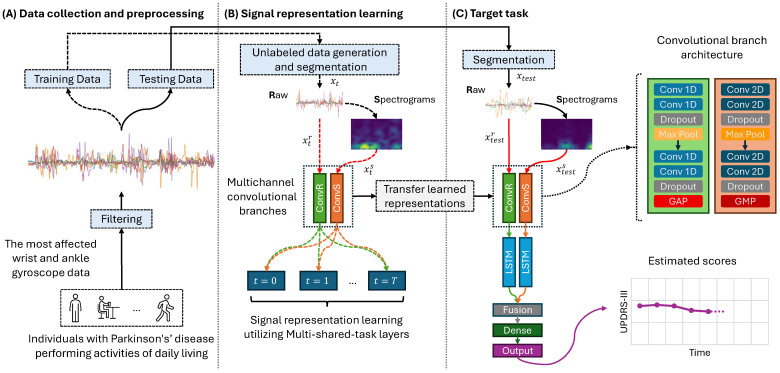
The architecture of our proposed model for estimating the UPDRS-III scores of PD patients. (**A**) outlines the data collection procedure and preprocessing steps. (**B**) depicts the signal representation learning stage, where the network undergoes various signal transformations to acquire robust features. (**C**) illustrates the UPDRS-III estimation network in the target, which incorporates the knowledge transferred from the signal representation learning network. GAP: global average pooling layer. GMP: global max pooling layer.

This section presents a detailed methodology outlining our approach to accurately estimating UPDRS-III scores for PD patients using wearable sensor data. Our investigation spans developing and integrating several deep neural network architectures, starting from simple models and advancing to more complex configurations. Initially, we created a 1D CNN to process raw gyroscope sensor data. This was followed by introducing a 2D CNN optimized to analyze spectrograms derived from gyroscope signal data. Building upon these initial models, we introduced a multichannel CNN configuration that processes raw sensor data and their corresponding spectrogram representations in parallel. This evolution of network architectures was followed by integrating CNNs with LSTMs, leading to the implementation of 1D, 2D, and multichannel CNN-LSTM networks. Such a comprehensive array of models allows for a robust framework that can capture the intricate spatial and temporal dynamics in the sensor data, enhancing the accuracy of UPDRS-III score estimation. We further enrich our methodology by introducing an innovative M-SSL CNN-LSTM model, showcasing our progressive exploration from simpler to more complex models. The details of each component and their integration into our unified approach are explained in the following sections. Figure 1 visually represents our study’s methodological flow and innovative aspects.

### 3.1. The Parkinson’s Disease Dataset

Motion data were captured from 24 individuals diagnosed with idiopathic PD as they engaged in various activities of daily living (ADL) following a carefully designed protocol [26,27]. A depiction of data collection and preprocessing is seen in Figure 1A. Among the participants, fourteen were female and ten were male, aged between 42 and 77 years, with disease durations ranging from 4 to 17 years. Before levodopa medication, the patients’ UPDRS-III scores spanned from 12 to 60, which reduced to a range of 4 to 38 after one hour of medication intake. Table 1 summarizes patients’ attributes. The study received approval from the Institutional Review Board at Rochester Medical Center in accordance with the Helsinki Declaration, and all participants provided written informed consent. Motion data were collected using two wearable sensors from Great Lakes NeuroTechnologies Inc., Cleveland, OH, USA equipped with a triaxial gyroscope and accelerometer positioned on the most affected wrist and ankle, capturing data at a sampling rate of 64 Hz. The anonymized collected data were provided to our team for further analysis.

**Figure 2:**
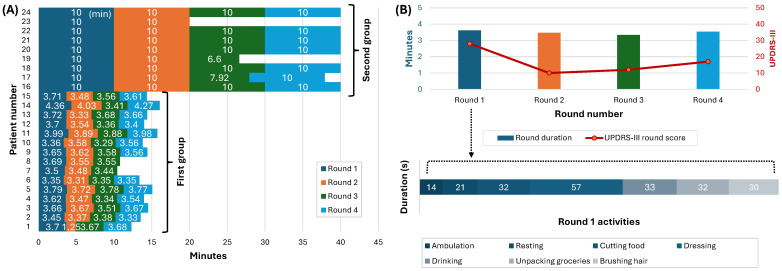
Data visualization from the study on 24 PD patients. (**A**) The duration (in minutes) and the number of rounds each participant completed. Each bar represents a round of data, with the height indicating the round’s duration. (**B**) A detailed breakdown of one participant’s activity duration across the four rounds. The red line shows the UPDRS-III scores in each round.

Participants were divided into two groups according to the data collection protocol, allowing the capture of a comprehensive range of ADLs under varied conditions. These activities were specifically selected to represent typical daily tasks, emphasizing the real-world applicability of our methodology for continuous UPDRS-III estimation in natural living environments. Figure 2A illustrates the participation and engagement of each of the 24 study participants in various rounds, providing a visual representation of the total duration spent by each individual in the study. As shown in this figure, 15 participants underwent four rounds of activities, which spanned over four hours, and 9 participants were involved in a continuous, two-hour session of unstructured, homelike activities. The ADLs for the first group included ambulation, resting, cutting food, dressing, drinking, unpacking groceries, and hygiene; each lasted between 15 and 60 s and was performed at the subjects’ self-paced rhythm without prior training. The ADLs for the second group were conducted in multiple stations and included laundry, watching television, snacking, and desk work, lasting about 10 min. The structure of the first protocol mirrored the daily variability in PD symptoms, considering the effects of medication over time. Conversely, the second group’s continuous session represented a snapshot of daily life challenges faced by individuals with PD for unobtrusive and continuous monitoring. Figure 2B offers a closer look into a detailed breakdown of one participant’s activity durations across the four rounds. It showcases the various tasks undertaken—from ambulation to more fine motor activities, like cutting food and dressing—and their respective durations.

All participants refrained from taking their PD medication the night before the experiment, initiating the trials in their medication OFF states, where the effects of the medication were minimal. After an initial round, participants resumed their PD medications, where the first group repeated the rounds every hour and the second group four times over two hours. A neurologist conducted clinical examinations to measure and record the participants’ UPDRS-III scores. Four rounds of UPDRS-III assessments were performed for the first group at the start of each experiment round. Two participants started the experiment in their medication ON state and had three assessment rounds. For the second group, two rounds of UPDRS-III assessments were conducted at the beginning and end of the experiment rounds. Due to some technical issues, twenty trials of activities were missing from the recordings of three subjects. The total number of the resulting round was 91 for all 24 participants. The red line in Figure 2B represents the UPDRS-III scores at each round.

### 3.2. Data Preprocessing

We opted for signals from the gyroscope sensors, which perform better than accelerometer sensors in estimating UPDRS-III scores [28]. To refine the data, we applied a bandpass Finite Impulse Response (FIR) filter with a 3 dB cutoff frequency of 0.5–15 Hz to eliminate low and high-frequency noise. Data collected during UPDRS-III examinations were excluded from the analysis to prevent the model from leveraging task-specific PD symptoms. Afterward, the signals were segmented into non-overlapping windows of 5 s durations to capture the characteristic symptoms of the disease [29]. Recognizing the spectral features of various PD symptoms, such as tremors in the 4–6 Hz range and bradykinesia in lower frequencies, we employed time-frequency representations of the signals to facilitate effective feature learning [24]. Accordingly, we generated corresponding spectrograms by applying a short-time Fourier transform (STFT) to the 5 s segmented windows, utilizing a 1 s Kaiser window with 0.9 overlaps.

### 3.3. The Utilized Deep Neural Networks Architectures

This subsection summarizes the fundamental framework of the 1D, 2D, multichannel CNN, and LSTM architectures, highlighting their configuration for processing raw gyroscope signals and their associated spectrograms.

#### 3.3.1. Convolutional Processing Branches

A CNN is a deep learning architecture commonly used for processing structured data arrays. CNNs are exceptionally proficient at signal processing tasks because they can automatically learn hierarchical patterns and features from input data [30]. In the context of our methodology, the CNN architecture consists of two convolutional branches:

- Raw Signal Branch (ConvR): This branch is responsible for processing the raw gyroscope signal component of the input, denoted as $x^{r}$. It employs 1D convolutional kernels in its layers, allowing the network to learn patterns directly from the raw signal data.
- Spectrogram Signal Branch (ConvS): In contrast, this branch processes the spectrograms generated from the input gyroscope signal, denoted as $x^{s}$. It utilizes 2D convolutional kernels in its layers to learn and extract features from the spectrograms, representing the signal’s frequency content over time.

#### 3.3.2. Multichannel CNN

A Multichannel CNN is an advanced architecture comprising multiple parallel convolutional layers with distinct kernel sizes. This design enables the simultaneous processing of input data through various filters, allowing the network to capture a broader range of features and patterns. Particularly effective for multi-input data streams, this approach enhances the network’s ability to discern complex patterns within the data [31]. In this study, we leverage the multichannel CNN approach to concurrently process the raw gyroscope signals $x^{r}$ and their corresponding spectrograms, $x^{s}$, using the ConvR and ConvS convolutional branches.

#### 3.3.3. LSTM Integration for Temporal Analysis

LSTM networks, subclasses of recurrent neural networks, address the vanishing gradient dilemma commonly encountered in training over long sequences. LSTM networks excel at recognizing long-term dependencies in data sequences, a trait crucial for tasks that rely on historical data patterns [32]. Integrating CNNs with LSTMs results in a CNN-LSTM architecture, which is adept at handling sequential data by utilizing CNN layers for initial feature extraction and LSTM layers for sequence modeling. This composite structure is particularly suitable for tasks requiring an understanding of spatial and temporal data dimensions [33].

### 3.4. Multi-Shared-Task Self-Supervised Learning

This subsection outlines the overarching structure of the multichannel CNN-LSTM, emphasizing its multi-branch design for learning representations from both the raw signals and their corresponding spectrograms in a self-supervised fashion using the proposed multi-shared-task learning approach.

We hypothesize that leveraging SSL in a novel multi-shared-task framework can significantly enhance the performance of deep learning models by acquiring meaningful data representations without the need for human annotation. SSL is a machine learning method that aims to enhance deep learning model performance by acquiring meaningful data representations without human annotation [34]. It operates through a two-stage learning process. First, the network tackles various signal transformation recognition tasks to learn robust features and signal representations from unlabeled data. Afterward, the pre-trained network from this phase is applied to the target task in the second stage, where the learned knowledge is transferred through transfer learning and fine-tuning techniques.

#### 3.4.1. Signal Representation Learning

In our work, to improve the monitoring of PD patients, we propose an innovative M-SSL methodology (see Figure 1B), building upon the previously introduced multi-task SSL [35]. This approach involves pre-training a multichannel CNN on various signal representations, leveraging unlabeled raw gyroscope signals $x^{r}$ and their spectrograms $x^{s}$ through the ConvR and ConvS convolutional branches. The outputs of the multichannel CNN branches are directed to the respective representation recognition task layers. The network incorporates a shared layer for each recognition task between the branches of the CNN, aligning with the correspondence of spectrograms and raw signals to the same data segment.

To train the network on various signal representations, we employed unlabeled data from the training set to generate signal transformations $x_{t}$ and corresponding pseudo labels $y_{t}^{p}$, where t=0,1,2,...,T represents the various signal transformations, and *T* is the number of these transformations. Thereon, spectrograms $x_{t}^{s}$ of $x_{t}$ were generated and concatenated with the raw signal $x_{t}^{r}=x_{t}$ to compose the network input $\left(\left[x_{t}^{r},x_{t}^{s}\right],y_{t}^{p}\right)$. A stochastic gradient descent method trains the network to recognize the *t* signal transformation, producing a probability $P_{t}$ indicating the likelihood of the signal being transformed from the original. The network’s total loss L is minimized by reducing the weighted average of individual losses corresponding to each signal transformation as follows:

(1) $$L=\sum_{t=0}^{T}\alpha_{t}\left[y_{t}^{p}\log\left(P_{t}\right)+\left(1+y_{t}^{p}\right)\log\left(1-P_{t}\right)\right],$$

where α represents the loss weight coefficients of the transformation tasks. We generated three signal transformations [36]:

- Rotation (t=1): This transformation involves applying a random rotation with an angle to the data to generate $\left(\left[x_{1}^{r},x_{1}^{s}\right],y_{1}^{p}\right)$. This enables the network to gain insights into different sensor placements.
- Permutation (t=2): This transformation randomly disrupts the temporal sequence within a data window by rearranging its segments, producing $\left(\left[x_{2}^{r},x_{2}^{s}\right],y_{2}^{p}\right)$. This allows the network to learn about the varying temporal positions of symptoms within the window data.
- Time warping (t=3): This transformation perturbs the temporal pattern of the data using a smooth warping path or a randomly located fixed window, which distorts the time intervals between samples and generates $\left(\left[x_{3}^{r},x_{3}^{s}\right],y_{3}^{p}\right)$. This method allows the network to learn about the changes in the temporal spacing of the samples.

These transformations are concatenated with the non-transformed (t=0) original signal $\left(\left[x_{0}^{r},x_{0}^{s}\right],y_{0}^{p}\right)$ to form the network input.

#### 3.4.2. Target Task: The Estimation of UPDRS-III Score

Following the signal representation learning stage using the proposed M-SSL, we transferred the knowledge acquired by the convolutional branches, ConvR and Conv.S, of the multichannel CNN, represented by the network weights, to the multichannel CNN component of the CNN-LSTM network, as depicted in Figure 1C. Afterward, the weights of the first convolutional blocks in the multichannel CNN-LSTM network were frozen, and the last convolutional blocks underwent fine-tuning using the annotated original training data, $\left(\left[x_{0}^{r},x_{0}^{s}\right],y\right)$, to estimate UPDRS-III scores. Estimating UPDRS-III scores involves regression, so we utilized the stochastic gradient descent algorithm to minimize the Huber loss function $L_{h}$ during the fine-tuning process. This loss function quantifies the error between the estimated $\hat{y}$ and clinical scores *y* as:

(2) $$L_{h}=\left\{\begin{array}{cc} \frac{1}{2}{\left(y-\hat{y}\right)}^{2}, & \;\mathrm{for}\;|y-\hat{y}|\;\leq \delta ,\\ \delta \cdot (|y-\hat{y}|-\frac{1}{2}\delta ), & \mathrm{otherwise}. \end{array}\right.$$

where δ=1 is where the loss function alternates between quadratic and linear behaviors. The decision to employ the Huber loss arose from its robust regression performance and reduced sensitivity to outliers within the data [37]. Following fine-tuning, the model was evaluated on the data from the testing set $\left(\left[x_{test}^{r},x_{test}^{s}\right],y_{test}\right)$, as depicted in Figure 1C.

### 3.5. Model Hyperparameters

#### 3.5.1. Signal Representation Learning Network

The proposed network to learn signal representations, as depicted in Figure 1B, consists of a multichannel CNN with the two branches ConvR and ConvS. ConvR processes the transformed raw gyroscope signal component, $x_{t}^{r}$. This branch comprises two 1D convolutional blocks with 64 kernels of size 32 in the first block and 128 kernels of size 8 in the second block. Following the first convolutional block, a max-pooling layer with a pooling size of 16 and strides of 4 is applied, and a global average-pooling layer follows the second block. Dropout rates of 0.1 and 0.2 are applied after each block. ConvS processes the generated spectrograms of the transformed signal, $x_{t}^{s}$, utilizing two 2D convolutional blocks. The first block contains 64 kernels of size 5×5, and the second has 128 kernels of size 3×3. Following the first convolutional block, the pooling size of the 2D max-pooling layer is 2 with strides of 2, and a global max-pooling layer follows the second block. A dropout rate of 0.1 is applied for each block. The size of the multi-shared-task layers is 128.

#### 3.5.2. UPDRS-III Estimation Network

The network for UPDRS-III estimation in the target task, as illustrated in Figure 1C, and the network for signal representation learning share the same multichannel CNN architecture. In addition to the convolutional branches, the UPDRS-III estimation network includes two LSTM layers, each with a size of 128 and a dropout rate of 0.1. The outputs of these layers are then fused and passed to a dense layer with a size of 256 before reaching the output layer, which estimates the UPDRS-III scores. This architecture is also shared across the series of deep neural networks discussed in Section 3.3. These hyperparameters were selected using a random 20% validation split of the training data, employing Bayesian optimization. The methodology’s Python code implementation is available on GitHub [38].

## 4. Results

**Figure 3:**
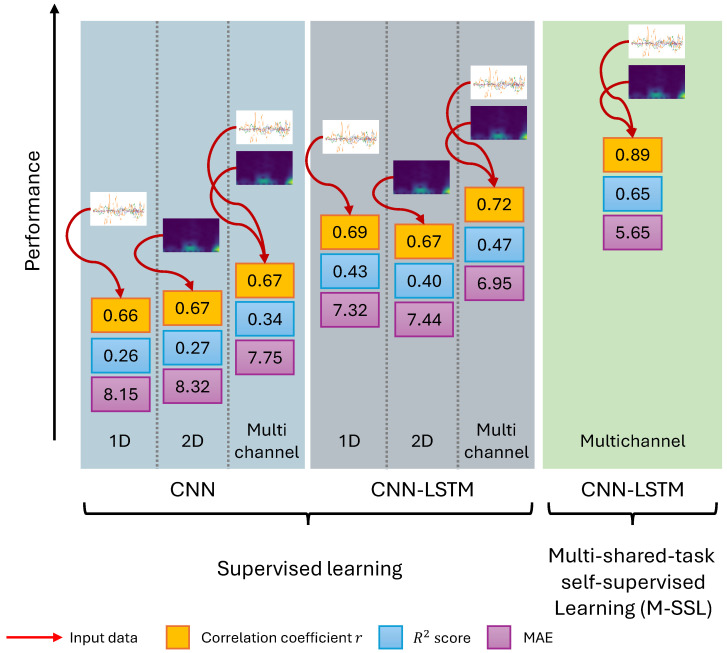
The proposed M-SSL multichannel CNN-LSTM performance metrics alongside the introduced supervised single and multichannel CNN and CNN-LSTM. The red arrows denote the model’s input data.

To evaluate the performance of our proposed M-SSL multichannel CNN-LSTM, we performed leave-one-out subject-wise testing utilizing all 24 subjects from both groups, where each subject was sequentially held out for testing while the remaining subjects were used for training. This process ensured that each subject acted as a test subject at least once. The signal representation learning and the UPDRS-III estimation networks were trained for 35 epochs with a batch size of 32, using the Adam optimizer with a 1 × $10^{-4}$ learning rate. Early stopping and learning rate scheduling strategies were employed to mitigate overfitting and stabilize the training process. We computed the mean estimated network scores for each UPDRS-III round and compared them to the clinical scores of the corresponding round. The practice of averaging the short-term estimated scores of PD symptoms over a longer duration has previously been adopted to smooth the effect of outliers in the model’s estimations [39]. The evaluation metrics included the correlation coefficient (*r*), coefficient of determination ($R^{2}$), and mean absolute error (MAE). The network configurations introduced in Section 3.3 were trained in a fully supervised setting on the labeled training data and applied to the testing data without learning signal representations. Figure 3 summarizes the average testing results for all 24 subjects and the improvements achieved with each model.

From Figure 3, we can observe that our proposed M-SSL multichannel CNN-LSTM surpasses all other supervised models by scoring the strongest correlation r=0.89$\left(p\leq 1\times 10^{-4}\right)$, indicating how well the model’s estimations approximate the actual clinical UPDRS-III score compared to other models. The performance is a substantial improvement from the r=0.72 achieved by the supervised multichannel CNN-LSTM and a significant one from the r=0.66 of the 1D CNN. Moreover, M-SSL multichannel CNN-LSTM exhibits the highest $R^{2}=0.65$ score among all networks. Further, the MAE reduces from 8.32 to 5.65. Another observation is that the multichannel CNN-LSTM outperforms CNNs and CNN-LSTMs when utilizing single input data, further underscoring the multichannel networks’ ability to capture complex data patterns effectively by combining raw sensor data and their spectrograms.

**Figure 4:**
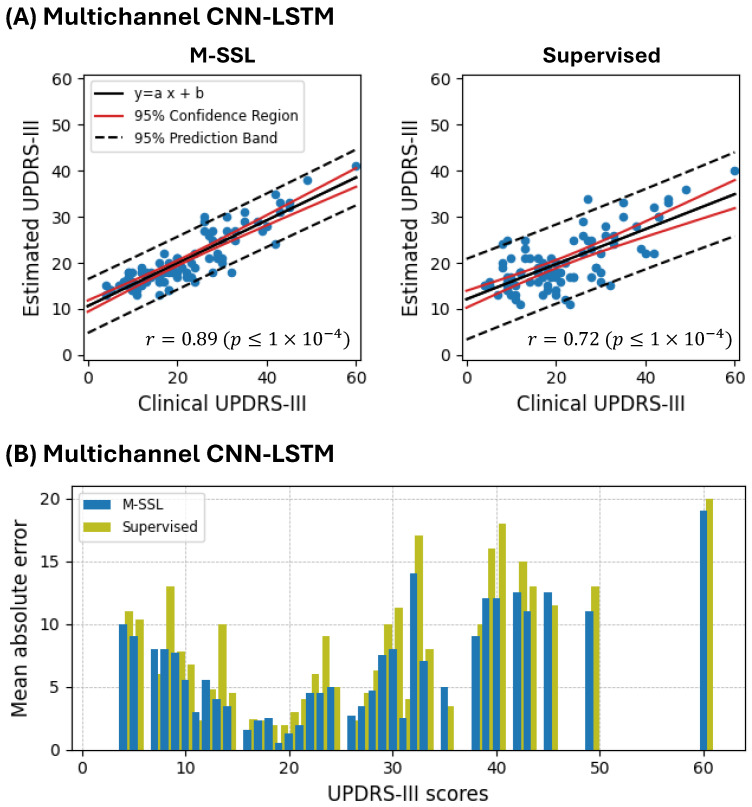
A performance comparison of estimating the UPDRS-III score between the proposed M-SSL and supervised multichannel CNN-LSTM. (**A**) depicts the clinical vs. estimated UPDRS-III scores for each round. (**B**) displays the mean absolute error for UPDRS-III scores.

**Figure 5:**
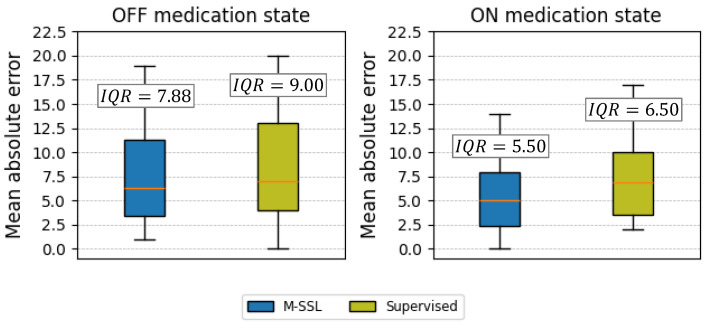
The mean absolute error of the estimated UPDRS-III scores before and after medication (OFF and ON medication states) using the proposed M-SSL and supervised multichannel CNN-LSTMs. The Interquartile Range (IQR) was calculated for each.

Figure 4A depicts the correlation between the clinical and estimated UPDRS-III scores for the M-SSL and supervised multichannel CNN-LSTM. Integrating the M-SSL strategy reduces the 95% confidence region and 95% prediction band in the multichannel CNN-LSTM, suggesting more precise estimations and enhanced performance. Furthermore, in Figure 4B, we observe that integrating M-SSL consistently reduces the MAE values across the various UPDRS-III scores. This observation underscores the M-SSL multichannel CNN-LSTM’s capability to accurately monitor PD patients with varying disease severity. We further explored the performance of M-SSL and supervised multichannel CNN-LSTM concerning patients’ medication states. As illustrated in Figure 5, the M-SSL approach enhances performance regardless of whether patients are ON or OFF their medications. Moreover, it maintains a more stable performance and less variability, as evidenced by the lower median and Interquartile Range (IQR) of the model’s MAE across all subjects and their UPDRS-III scores.

**Figure 6:**
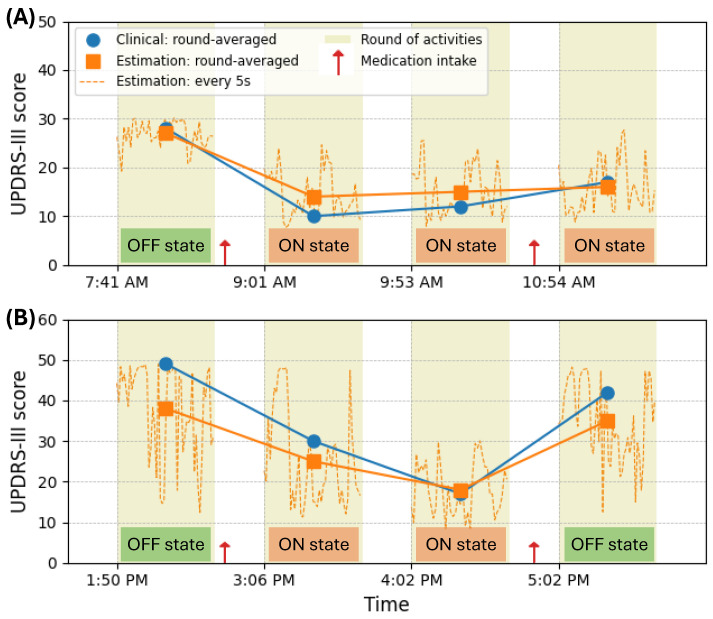
The UPDRS-III estimations over time by the proposed M-SSL multichannel CNN-LSTM compared to the clinical UPDRS-III scores for two PD patients. (**A**) illustrates a patient showing improvement in PD symptoms over time. (**B**) displays a patient experiencing the return of PD symptoms before the next dose of medication.

Two examples of the proposed M-SSL multichannel CNN-LSTM’s estimation of UPDRS-III scores are illustrated in Figure 6. These examples display the estimations at 5 s windows, the round-averaged estimations, the rounds, and the clinically documented scores provided by the neurologists for each round. In Figure 6A, the subject initiated the experiment in their medication OFF state with a recorded UPDRS-III of 27. Following medication, the subject transitioned and remained in the medication ON state for the subsequent three rounds, with scores of 14, 15, and 16. In Figure 6B, the subject exhibited more severe symptoms at the experiment’s onset, with a UPDRS-III score of 49. Symptoms improved after medication intake for rounds 2 and 3. However, in round 4, the patient experienced a deterioration in disease severity upon transitioning into the OFF state, registering a score of 42. The proposed model consistently estimated disease severity across varying symptom levels in both cases, accurately capturing changes throughout the experiment.

**Figure 7:**
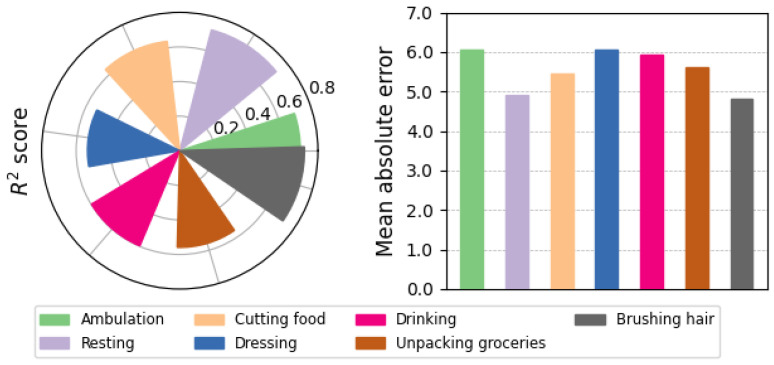
The coefficient of determination $R^{2}$ and the mean absolute error of the UPDRS-III estimations by the proposed M-SSL multichannel CNN-LSTM during patients’ activities.

We also evaluated the performance of M-SSL multichannel CNN-LSTM in estimating the severity of motor symptoms during different activities, as depicted in Figure 7. We calculated the $R^{2}$ and MAE scores for the model’s estimations during each daily living activity. The results indicate that the model’s performance remained consistent across various activities, with $R^{2}=0.64\pm 0.07$ and MAE=5.55±0.48, demonstrating robustness to changes in activity while estimating UPDRS-III scores for PD patients.

## 5. Discussion

In the growing field of PD research, accurately monitoring disease severity and progression presents a significant challenge. We introduced several deep neural network architectures to tackle this challenge. We explored their performance in a PD dataset to estimate the UPDRS-III score in individuals with PD using data from wearable sensors during unobstructed free-body daily activities. The continuum of deep neural network architectures introduced in our study yielded a progressive enhancement in performance, culminating in the M-SSL multichannel CNN-LSTM architecture demonstrating the highest effectiveness.

This achieved progressive enhancement is attributable to various vital factors. First, our model leveraged a deep CNN architecture, demonstrating promising results in estimating PD motor severity from raw signal data collected by wearable sensors [25]. Additionally, by incorporating signal spectrograms, our approach effectively extracted features from the temporal and spectral characteristics of the signals. This strategy, particularly evident in the improved performance of the 2D CNN $\left(r=0.67,R^{2}=0.27\right)$ compared to the 1D CNN $\left(r=0.66,R^{2}=0.26\right)$, has been shown to enhance UPDRS-III score estimation [24], given that PD symptoms often manifest spectral features, like tremors and bradykinesia.

Furthermore, integrating a multichannel CNN in our methodology enhanced the network’s capacity to learn and extract richer features from the raw and frequency representations of multi-input signals, as demonstrated by its $\left(r=0.67,R^{2}=0.34\right)$. This approach has been explored previously and has shown benefits in processing wearable data [40]. Further, incorporating LSTM layers alongside the CNN enabled the model to capture temporal dependencies in the input data. This is apparent in the improved performance of the 1D $\left(r=0.69,R^{2}=0.43\right)$, 2D $\left(r=0.67,R^{2}=0.40\right)$, and multichannel CNN-LSTM $\left(r=0.72,R^{2}=0.47\right)$ over their respective CNN counterparts. The effectiveness of the CNN-LSTM architecture in processing wearable sensor data has been well documented [41].

Another significant contributing factor to the robust performance of our methodology is the signal representation learning phase, where the model was trained in a self-supervised manner to learn various data representations. This process exposed the model to signal transformations such as rotation, permutation, and time warping. Previous studies, such as [36], have shown that augmenting a CNN with these transformations can enhance its performance in monitoring PD symptoms. These transformations effectively address sensor position variability and the temporal variability of events within the collected PD data. By incorporating signal representations from these three transformations, our model further improved its UPDRS-III estimation performance. This enhancement, coupled with the innovative M-SSL approach employed in our methodology, underscored the effectiveness of our strategy in achieving superior estimation performance with a correlation coefficient $r=0.89(p<1\times 10^{-4})$, coefficient of determination $R^{2}=0.65$, and MAE of 5.65.

A comparison of recent state-of-the-art studies utilizing machine learning and wearable sensors to estimate UPDRS-III scores in patients diagnosed with PD is presented in Table 2. Our proposed M-SSL multichannel CNN-LSTM model exhibits superior performance compared to other approaches, offering additional benefits such as activity flexibility (non-task-dependent), a broader range of activities, automated feature extraction (eliminating the need for manual feature engineering), and minimal sensor requirements. For instance, our method outperformed the approach by Zhan et al. [20] (r=0.89 vs. 0.81), despite their methodology requiring patients to perform five specific tasks using a smartphone application and employing feature extraction techniques. Similarly, the proposed M-SSL multichannel CNN-LSTM surpassed the performance achieved by Butt et al. [21] (r=0.89 vs. 0.81), which involved patients performing UPDRS-III-specific tasks and feature extraction. Additionally, our approach showed more robust estimation capability than Sotirakis et al. [22] (RMSE =6.92 vs. 10.02), where patients performed walking and postural sway tasks; then, features were extracted from six wearable sensor data. Furthermore, a study by Rehman et al. [25] was inferior (r=0.89 vs. 0.82) despite focusing solely on patients performing walking tasks. In addition to the aforementioned comparisons, we directly compared our proposed M-SSL multichannel CNN-LSTM model with the approaches by Hssayeni et al. [24] and Rehman et al. [25], as they also utilized raw sensor data similar to our methodology. Our M-SSL multichannel CNN-LSTM model exhibited superior UPDRS-III monitoring performance, achieving respective correlations of (r=0.89 vs. 0.74 vs. 0.69).

While our proposed algorithm has demonstrated outstanding performance, it is essential to acknowledge certain limitations. One observation is that the model underestimates high UPDRS-III scores, as depicted in Figure 4A. This discrepancy can be attributed to the imbalanced data distribution, particularly concerning higher UPDRS-III scores. For instance, there is only one round of activities with a UPDRS-III score higher than 50. Previous studies have reported similar behavior, such as that by Parisi et al. [42]. Therefore, our future work will focus on collecting more data from the PD population with diverse disease severity. Moreover, improving the interpretability of our deep learning model could involve incorporating techniques such as attention mechanisms and gradient-based methods.

Responding to clinical feedback and recognizing the complexities of neurodegenerative disease monitoring, we have incorporated a broader symptomatic assessment within our study; while our wearable sensors are optimized for capturing detailed motion data and do not directly measure speech clarity or facial expressions as per UPDRS-III items 18 and 19, these dimensions are integral to understanding the full spectrum of disease impact. This comprehensive approach allows us to contextualize the quantitative data from the wearables within a broader clinical framework, thereby providing deeper insights into patient symptoms. Future iterations of this research will aim to integrate additional sensor technologies to capture these and other vital aspects, enhancing the holistic assessment capabilities of our monitoring tools. This study utilized the UPDRS Part III due to the specific dataset, which was collected before the widespread adoption of the MDS-UPDRS. Notably, there is a strong correlation between UPDRS and MDS-UPDRS scores, ensuring the continued applicability of our findings [43,44]. Recognizing the benefits of the MDS-UPDRS’s enhanced specificity, we plan to incorporate this updated tool in our future research efforts.

This work’s significance in bioengineering lies in its potential to improve PD monitoring and management immensely. The ability to accurately monitor disease progression and symptom severity in real-time, without frequent clinical visits, can empower clinicians with timely and accurate information for personalized treatment planning and disease management in PD patients.

## 6. Conclusions

Our innovative M-SSL approach, implemented through a multichannel CNN-LSTM network, efficiently processes and analyzes motion data to capture the complexity of PD symptoms in free-body, unconstrained settings to estimate UPDRS-III scores in PD patients. The methodology integrates signal transformations such as rotation, permutation, and time warping to enhance the network’s learning capability and accuracy. The experimental results from 24 PD subjects with a data duration of 526 min demonstrate the efficacy of our approach, achieving a high correlation of r=0.89 between the estimated and clinically documented UPDRS-III scores. This performance surpasses traditional 1D, 2D, and multichannel CNN and CNN-LSTM models and outperforms other recent state-of-the-art methods, establishing a new benchmark in the field. Such findings underscore the potential of our method to improve PD monitoring significantly, providing clinicians with a reliable and objective tool to assess disease severity and optimize treatment strategies. Furthermore, our proposed methodology has broad potential applications within bioengineering, providing pathways for addressing challenges across various medical conditions and paving the way for future innovations in patient care and disease management that underscore the critical role of engineering solutions in improving healthcare outcomes.

## Figures and Tables

**Table 1:** The participants’ demographics.

Participant Attributes	Value/Mean ± std
Total number	24
Sex (male, female)	14,10
Age (years)	58.8±9.5
Disease duration (years)	9.9±3.8
UPDRS-III prior to medication	30.3±11.6
UPDRS-III after medication	16.4±8.4
Levodopa equivalent daily dose LEDD (mg)	1251±478

**Table 2:** Recent UPDRS-III estimation methods in the literature.

Method	Dataset	Sensors No.	Method’s Input	Activities	*r*	$R^{2}$	MAE	RMSE
Zhan et al. [20]	Theirs	1	Features extracted from smartphone data	5 smartphone tasks	0.88	−	−	−
Butt et al. [21]	Theirs	2	Features extracted from accelerometer and gyroscope	12 MD-UPDRS-III-specific tasks	0.81	−	−	−
Sotirakis et al. [22]	Theirs	6	Features extracted from accelerometer and gyroscope	Walking and postural sway	−	−	−	10.02
Rehman et al. [25]	Theirs	1	Accelerometer raw	Walking	0.82	−	−	−
Hssayeni et al. [24]	Ours	2	Gyroscope raw and spectrograms	7 ADL	0.74	0.51	6.54	8.19
Rehman et al. [25]	Ours	2	Accelerometer raw	7 ADL	0.69	0.40	7.35	9.05
Proposed M-SSL multichannel CNN-LSTM	Ours	2	Gyroscope raw and spectrograms	7 ADL	0.89	0.65	5.65	6.92

## Data Availability

The data presented in this study are available upon request from the corresponding author due to privacy constraints.

## Abbreviations

The following abbreviations are used in this manuscript:

PD	Parkinson’s disease
UPDRS	The Unified Parkinson’s Disease Rating Scale
CNN	Convolutional Neural Network
LSTM	Long Short-Term Memory
SSL	Self-supervised Learning
M-SSL	Multi-shared-task Self-supervised Learning
ADL	Activities of Daily Living
FIR	Finite Impulse Response
MAE	Mean Absolute Error
IQR	Interquartile Range
